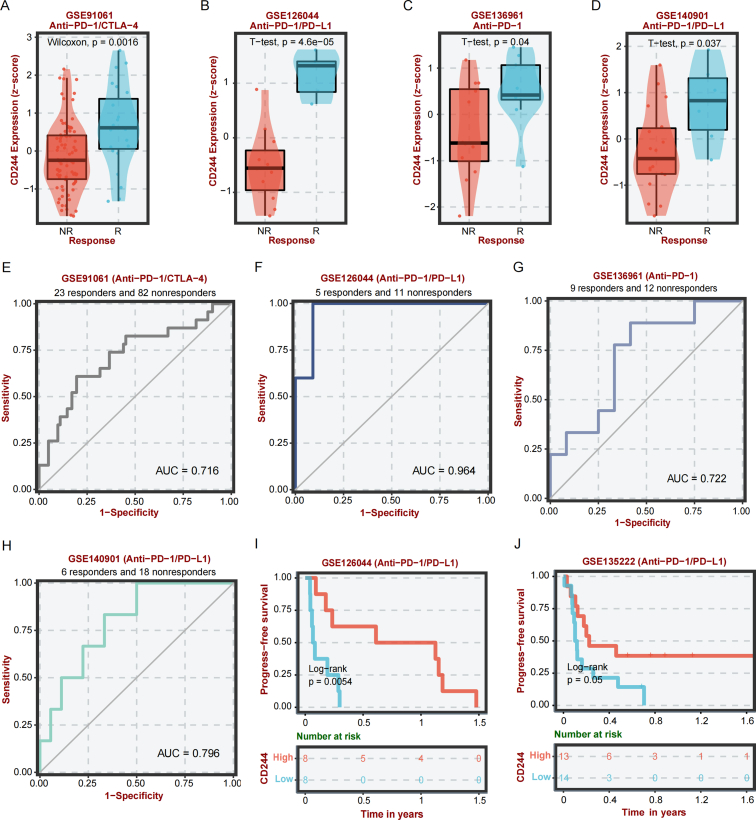# Distinct roles of CD244 expression in cancer diagnosis and prognosis: A pan-cancer analysis

**DOI:** 10.1016/j.heliyon.2024.e28928

**Published:** 2024-03-30

**Authors:** Zhenzhen Deng, Yuanhong Liu, Haiyan Zhou

**Affiliations:** a Department of Pharmacy, The Third Xiangya Hospital, Central South University, Changsha, 410008, Hunan, China; b Department of Pharmacy, Xiangya Hospital, Central South University, Changsha, 410008, Hunan, China; c Department of Pathology, School of Basic Medicine, Central South University, Changsha, Hunan, China

**Keywords:** CD244, Pan-cancer, Biomarker, Immune infiltration, Immunotherapy target, Prognosis

## Abstract

The abnormal expression of tumor associated genes in pan-cancer is closely related to the clinicopathological features of distinct cancer types. Thus, identifying the role of specific genes in pan-cancer is needed for developing effective anti-cancer strategies. However, the function of CD244 in pan-cancer has not been fully understood. In this study, we explored the CD244 expression profile across 33 tumor types based on The Cancer Genome Atlas project, the Gene Expression Omnibus database, and other bioinformatics tools. We found down-regulated expression levels in seven tumor types and up-regulated expression levels in two tumor types. We subsequently explored the relationship between survival rate and CD244 expression, and found the positive relationship in patients with adrenocortical carcinoma (ACC), head and neck squamous cell carcinoma (HNSC), skin cutaneous melanoma (SKCM), and uterine corpus endometrial carcinoma (UCEC). We further investigated the association between CD244 expression and tumor-infiltrating immune cells, and discovered their positive correlation in different tumors. We found that CD244 expression level was higher in normal samples than in UCEC samples, and was positively associated with CD8^+^ T cells infiltrating. The mutation status, promoter methylation, CD244-related molecules and signaling pathways were also employed to study the potential function of CD244 in tumor initiation and progression. Our study offers a comprehensive overview of CD244 in human tumors, revealing CD244 as a potential prognostic biomarker and immunotherapeutic target in cancers.

## 1 Introduction

In recent years, the emergence and advancements of immunotherapeutic strategies (such as immune checkpoint blockade) have provided novel strategies and drugs for cancer treatment [1,2]. However, due to the complex pathogenic mechanism and immunity of tumors, a considerable proportion of patients still do not benefit from these therapeutic agents [3,4]. Thus, determining novel therapeutic targets and prognostic biomarkers to ameliorate the outcome of patients with cancer is of utmost importance.

CD244 (leukocyte differentiation antigen 244), also known as 2B4 and SLAMF4 (the signaling lymphocyte activation molecule family 4), is one of the immunoglobulin superfamily molecules [5]. It acts as an immunomodulatory transmembrane receptor mainly expressed on the surface of all T and NK cells that mediate non-MHC-restricted killing [6]. It was also found on dendritic cells and other immune cells [7]. CD244 primarily binds to its high-affinity ligand CD48 to transmit stimulatory or inhibitory signals [8,9]. Moreover, CD244 expressed on one NK cell interacts with CD48 on neighboring cells (in trans) and with CD48 on the same cell (in cis) [10]. Interaction in cis decreases the ability of CD244 to bind to CD48 in trans. CD244 plays a critical role in regulating immune responses such as NK cell-mediated cytotoxicity, leukocyte activation, cytokine production, and clearance of tumor cells [11,12]. Previous studies have shown that CD244 is related to many immune-related diseases, involving cancers and infectious diseases, and is involved in their onset and progression [5,[13], [14], [15]]. With recent in-depth research, studies revealed that CD244 is a promising biomarker and immunotherapy target in some cancers, such as lung cancer [16]. However, a comprehensive pan-cancer analysis regarding the roles of CD244 across other cancer types is not yet clear.

Pan-cancer analysis can more systematic understanding of the common and distinct molecular features in distinct tumor types [17]. It involves biomarkers, prognosis, genetic alterations, DNA methylation, tumor immune infiltration, immunotherapy, and others. It is a prospective research method for identifying novel biomarkers and formulating treatment strategies on the basis of these specific biomarkers.

In our present study, we conducted a comprehensive pan-cancer analysis on the roles of CD244 expression (Supplementary Fig. 1). We explored the CD244 expression profile across different tumors based on the TCGA and GEO database. Moreover, we investigated the relations between the expression level of CD244 and the survival rate of patients with cancer. In addition, we performed immunohistochemistry (IHC) to confirm the CD244 expression in uterine corpus endometrial carcinoma (UCEC). We also detected a positive correlation of CD244 expression with CD8^+^ T cells infiltrating in UCEC. We also studied the genetic mutations of CD244, CD244 promoter methylation level, tumor functional status related to CD244, and CD244 related co-expression molecules and related signaling pathways. Our findings revealed that CD244 has the potential to be as a new prognostic biomarker.

## 2 Material and methods

### 2.1 CD244 gene expression across different tumor types

We downloaded the pan-cancer datasets (including TCGA and GTEx datasets, N = 19131) from the UCSC database, extracted data of CD244 expression in every single sample type, and converted these expression values into the log2(TPM+1) format. Then, we analyzed the differences in expression between tumors and normal samples in every tumor type with SangerBox 3.0 (http://vip.sangerbox.com/home.html), an open-access online bioinformatics analysis tool. We analyzed significance in comparisons using the Wilcoxon rank sum and signed rank tests. We subsequently used the TCGA dataset alone to compare the expression levels between tumor and normal samples through the Xiantao tool (https://www.xiantaozi.com/). Based on the original data from the TCGA program, we further analyzed and verified the gene expression level of CD244 in different tumor types and corresponding adjacent normal tissues using TIMER2.0 (Tumor Immune Estimation Resource, version 2.0) [18,19].

Furthermore, to explore the expression level of CD244 in different pathological stages of all TCGA tumors, we obtained violin plots concerning the relationship between gene expression and pathological stages using the TISIDB database [20]. We used the major stage for plotting and log_2_ (CPM) for log-scale.

### 2.2 CD244 related survival and prognosis analysis

GEPIA2 (Gene Expression Profiling Interactive Analysis, version 2) was utilized to assess the overall survival (OS) and disease-free survival (DFS) related to the CD244 expression in all TCGA cancer types [21]. We selected the median expression of CD244 as the cutoff value to categorize cancer samples into the high-expression and low-expression groups. The log-rank test was used as hypothesis test to analyze the relationship between the distinct expression levels of CD244 and the survival rate of various tumors, with a 95% confidence interval and the hazard ratios (HR) were deduced based on Cox PH model.

Meantime, we used TIMER2.0 to confirm the correlation between the expression of CD244 and survival in various tumor types. Furthermore, we downloaded the RNA-sequencing expression profiles and corresponding clinical information related to CD244 from the TCGA dataset in order to perform the univariate and multivariate COX regression analysis of OS for UCEC using the Xiantao tool. P < 0.05 was considered statistically significant.

### 2.3 CD244 genetic alterations and mutations analysis

We utilized the cBioPortal tool to collect data regarding CD244 mutation site, mutation type, alteration frequency, and copy number alteration (CNA) across all TCGA tumors [22,23]. Survival probability (OS and DFS) between the altered and unaltered groups were compared. Log-rank test was used for the hypothesis test, and the p < 0.05 was regarded as statistical significance.

### 2.4 CD244 promoter methylation analysis

The UALCAN tool was exploited to analyze the CD244 promoter methylation profile for different primary tumors and corresponding normal tissues in order to evaluate epigenetic regulation of gene expression by promoter methylation [24,25].

### 2.5 Immune infiltration analysis and immunohistochemical staining

We downloaded all correlated data of immune infiltration across TCGA cancers from the TIMER2.0 website. B cell, T cell CD8^+^, dendritic cell, neutrophil, monocyte, NK cell, and macrophage were selected in the analysis of the correlation between CD244 expression and immune infiltration using QUANTISEQ, EPIC, TIMER, CIBERSORT, CIBERSORT-ABS, MCPCOUNTER, XCELL, and TIDE algorithms. Meanwhile, we used the TISIDB database for further exploration.

Then, we downloaded RNA sequencing data of UCEC in FPKM (Fregments Per Kilobase per Million) format based on the program level 3 of the TCGA database. Subsequently, we calculated the correlation between CD244 expression and immune infiltration cells using the Xiantao tool. According to the afore-mentioned analysis, we obtained the correlation data of CD244 expression in UCEC and immune infiltration cells as well as immune checkpoints using the TISIDB and GEPIA2 databases. Furthermore, eight tissue samples of UCEC from DISCO database [26] were used to analyze the relationship between CD244 and immune infiltrating at single cell level. Moreover, we used BEST database [27] to explore the relationship between CD244 expression and immunotherapy response in cancer patients.

Besides, IHC was operated to confirm the relationship between CD244 expression and immune cell infiltration. The materials used were as follows: CD8 monoclonal antibody (Cat No.66868-1-Ig 50 μL), CD244 polyclonal antibody (Cat No.16677-1-AP 50 μL), and tissue microarray of UCEC (HUteA060CS01). IHC intensity score was scored as 0 (negative), 1 (weak brown), 2 (moderate brown), or 3 (strong brown). The extent of staining was scored as 0 (≤10%), 1 (11%–25%), 2 (26%–50%), 3 (51%–75%), or 4 (>75%). The final staining score was determined by multiplying intensity and extent scores. All paraffin-embedded specimens were collected following the ethical standards of the human experimental committee and were approved by the Ethics Committee of Shanghai Outdo Biotech Company (Ctl No. YBM-05-02).

### 2.6 Single-cell sequencing data analysis

We downloaded data related to CD244 expression and functional status in various tumors based on single-cell sequencing data from CancerSEA [28]. The CancerSEA is a professional database for single-cell sequencing and provides an opportunity to assess the functional statuses of cancer cells at the single-cell level. Subsequently, we plotted a correlative heatmap using the Xiantao tool. Besides, the distribution of CD244 expression in particular cells was displayed by t-SNE diagrams that were acquired directly from the CancerSEA website.

### 2.7 CD244 related gene functional enrichment analysis

We analyzed the proteins interacting with CD244 through the STRING website (version 11.5) [29]. The following parameters were set to obtain the proteins binding to CD244: 1) full STRING network for network type; 2) experiments for active interaction sources; 3) low confidence (0.150) for the minimum required interaction score. The "meaning of network edges" was definite as evidence, and the "max number of interactors to show" was definite as no more than 50 interactors. Moreover, we also assembled the genes with similar expression patterns to CD244 based on all TCGA tumors using GEPIA2. The top 100 similar genes were considered as candidate genes for the subsequent analysis. GO (Gene Ontology) enrichment analysis and KEGG (Kyoto Encyclopedia of Genes and Genomes) pathway analysis of CD244 and its binding proteins were completed using Xiantao tool.

## 3 Results

### 3.1 CD244 gene expression data in different tissues and clinicopathological stages

In this section, we explored the expression status of CD244 in normal and tumor samples with various pathological stages. As revealed in Fig. 1A–C, among these tumor types, including UCEC, colon adenocarcinoma (COAD), liver hepatocellular carcinoma (LIHC), prostate adenocarcinoma (PRAD), lung adenocarcinoma (LUAD), rectum adenocarcinoma (READ), and lung squamous cell carcinoma (LUSC), CD244 expression were significantly lower in the tumor tissues than in the normal tissues. However, the expression levels of CD244 in tumor tissues were up-regulated in kidney renal papillary cell carcinoma (KIRC) and kidney renal papillary cell carcinoma (KIRP). No significant difference was discovered in other TCGA tumors.

**Fig. 1:**
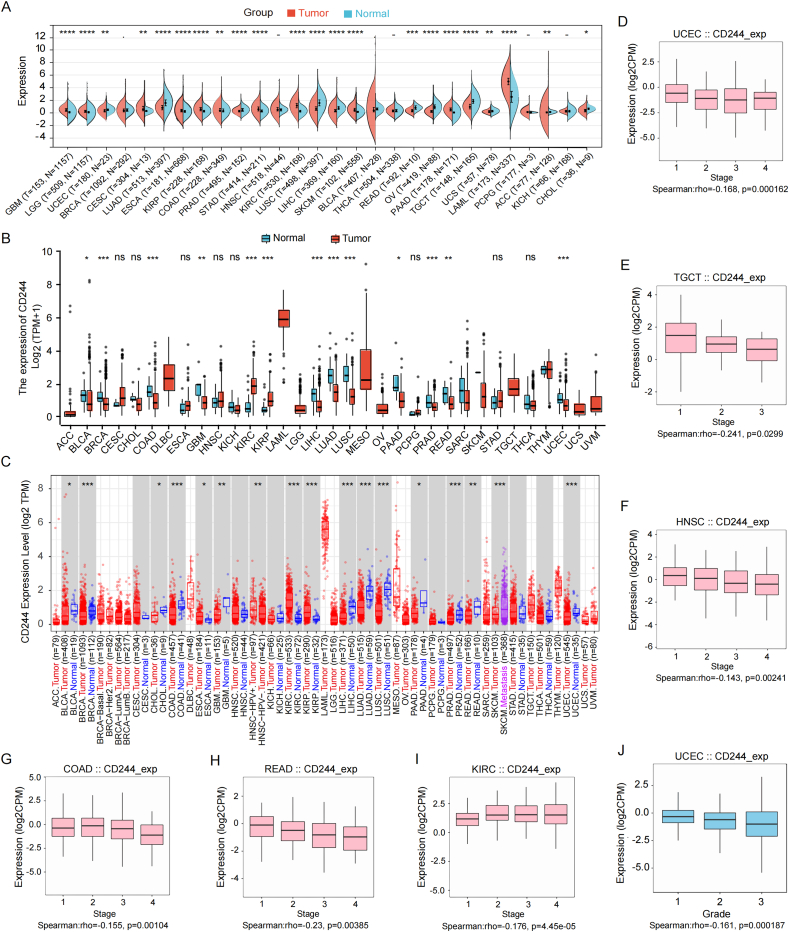
Variable expression of CD244 and its relationships with clinicopathological stages across TCGA tumors. (A) CD244 expression levels among tumors and adjacent tissues based on TCGA and GTEx databases (****p < 0.0001; ***p < 0.001; **p < 0.01; *p < 0.05). (B) CD244 expression levels among tumors and adjacent tissues based on TCGA database. (C) CD244 expression levels among tumors and adjacent tissues based on TCGA database were assessed by TIMER2.0. (D–J) Correlation between CD244 expression and clinicopathological stages/grade across TCGA tumors according to the TISIDB database. UCEC, TGCT, COAD, HNSC, KIRC, and READ show statistically significant results. Gene expression levels are presented as Log_2_ (CPM).

Meanwhile, TISIDB was employed to investigate the relationship between CD244 expression levels and pathological stages. The results demonstrated a negative correlation between the clinical-pathological stages and CD244 expression levels in UCEC, testicular germ cell tumors (TGCT), READ, COAD, and head and neck squamous cell carcinoma (HNSC) (Fig. 1D–H). Instead, the pathological stages of KIRC revealed a positive correlation with CD244 expression(Fig. 1I). In other TCGA tumor types, there was no correlation between the pathological stage and CD244 expression level (Supplementary Fig. 2A–S). Notably, we also found that the histological grade of UCEC was negatively linked with the expression level of CD244 (Fig. 1J).

### 3.2 The prognostic values of CD244 in patients

We mainly intended to determine whether the CD244 expression was relevant to tumor survival time. OS and DFS relative to CD244 in 33 tumor types were assessed using GEPIA2 (Fig. 2A and B). According to the survival data conducted by TIMER2.0, there was an association between elevated CD244 expression and favorable prognosis in HNSC, SKCM, and UCEC (Fig. 2C). Then, through the 'forest plot' R package, we found that CD244 could be as a protective factor in multiple tumor types, such as adrenocortical carcinoma (ACC), HNSC, skin cutaneous melanoma (SKCM) and UCEC (Fig. 2D). Multivariate analysis indicated that the OS in UCEC was largely associated with clinical stage, histological grade, and effective radiotherapy (Table 1).

**Fig. 2:**
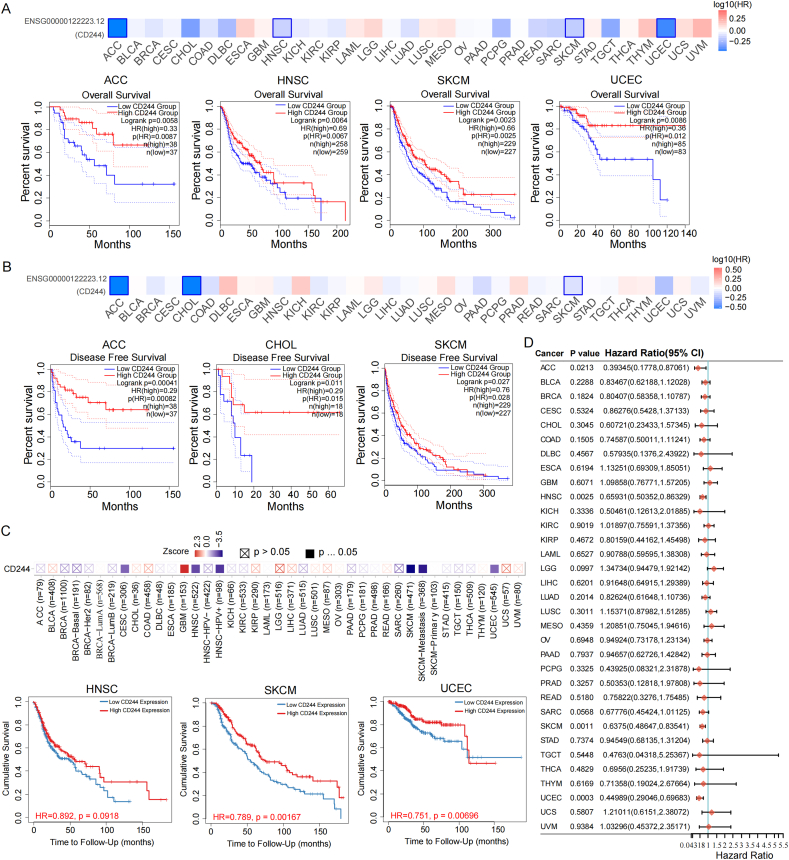
Prognosis related to CD244 expression in tumors. Overall survival (A) and disease-free survival (B) in different tumor types were respectively assessed. Survival differences among the high and low expression cohorts were analyzed. HR illustrates a hazard ratio of the high-expression sample relatives to the low-expression sample. HR > 1 shows the gene functions as a risk factor, and HR < 1 offers a protective one. (C) TIMER2.0 was applied for the survival analyses in TCGA cancers. (D) Forest plot indicates p-value, risk coefficient (HR), and prognostic characteristics of CD244 from the single factor cox analysis of TCGA tumors.

**Table 1:** An univariate and multivariate analysis of risk factors and OS in UCEC.

Variables	HR (95% CI)	P-value
Univariate analysis		
CD244(low vs. high)	0.645 (0.433–0.961)	0.031
Clinical stage (Stage I &Stage II vs. Stage III &Stage IV)	3.543 (2.355–5.329)	<0.001
Primary therapy outcome (PD&SD vs. PR&CR)	0.139 (0.079–0.243)	<0.001
Race (Asian vs. White)	2.854 (0.698–11.674)	0.145
Age (≤60 years vs. >60 years)	1.847 (1.160–2.940)	0.010
Histologic grade (G1&G2 vs. G3)	3.281 (1.907–5.643)	<0.001
Tumor invasion (<50% vs.≥50%)	0.896 (0.487–1.649)	<0.001
Menopause status (Pre &Peri vs. Post)	1.050 (0.507–2.175)	0.895
Residual tumor (R0 vs. R1&R2)	3.101 (1.768–5.440)	<0.001
Hormones therapy (No vs. Yes)	0.801 (0.380–1.689)	0.560
Radiation therapy (No vs. Yes)	0.594 (0.385–0.915)	0.018
Multivariate analysis		
CD244(low vs. high)	0.682 (0.368–1.263)	0.224
Clinical stage (Stage I &Stage II vs. Stage III &Stage IV)	4.317 (2.061–9.040)	<0.001
Primary therapy outcome (PD&SD vs. PR&CR)	0.474 (0.164–1.368)	0.167
Residual tumor (R0 vs. R1&R2)	1.980 (0.783–5.003	0.149
Histologic grade (G1&G2 vs. G3)	1.730 (0.845–3.540)	0.134
Tumor invasion (<50% vs.≥50%)	1.192 (0.588–2.416)	0.626
Radiation therapy (No vs. Yes)	0.388 (0.206–0.732)	0.003

OS, overall survival; HR, hazard ratio; CI, confidence interval; PD, progressive disease; SD, stable disease; PR, partial response; CR, complete response.

### 3.3 CD244 genetic alterations and mutations analysis data

The changes of tumor suppressor genes or oncogenes could cause abnormal signaling pathways, affecting anomalous cell proliferation, growth, differentiation, and cancer metastasis [30]. Therefore, we intended to further explore the genetic mutations or alteration of CD244 in various cancers to outline its roles in human tumors. The maximum alteration frequency of CD244 in cancers was above 15%, in which "amplification" is the primary type of genetic alteration, followed by "mutation" (Supplementary Fig. 3A). The tumors with more than 5% alteration frequency were bladder urothelial carcinoma (BLCA), cholangiocarcinoma (CHOL), LIHC, breast invasive carcinoma (BRCA), lung adenocarcinoma (LUAD), UCEC, SKCM, LUSC, and sarcoma (SARC). Among all mutation sites of CD244, the major type of genetic alteration was "missense" (Supplementary Fig. 3B). We then compared the survival data of altered and unaltered groups to explore the prognostic roles of CD244 genetic alteration. Our findings revealed that the unaltered group had a better survival prognosis than the altered group (Supplementary Fig. 3C–E).

### 3.4 CD244 promoter methylation

Furthermore, we compared the CD244 promoter methylation levels in distinct tumor types with those in corresponding normal tissues. As implied in Fig. 3, LUAD and LUSC showed higher levels of CD244 methylation than that in normal tissues. In contrast, other tumors such as PCPG, LIHC, KIRP, glioblastoma multiforme (GBM), COAD, BLCA, SARC, prostate adenocarcinoma (PRAD), BRCA, thyroid carcinoma (THCA), UCEC, HNSC, and TGCT showed relatively lower methylation levels of CD244. No significant differences about CD244 methylation levels could be found in the remaining tumor samples (Supplementary Fig. 4).

**Fig. 3:**
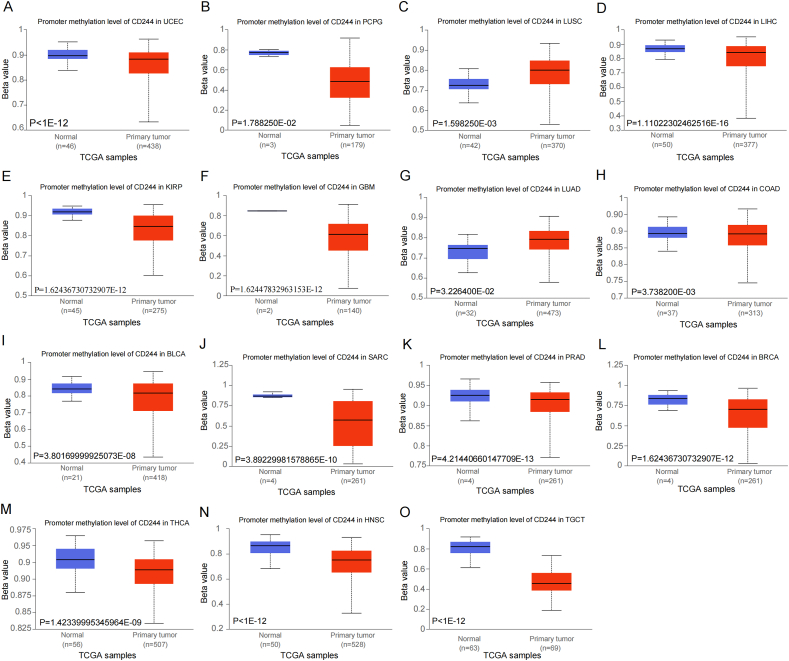
Promoter methylation levels of CD244 in different primary tumor samples and the corresponding normal counterparts. The Beta-value means that the CD244 methylation level ranges from unmethylated (0) to fully methylated (1). Hyper-methylation reflects the beta-value ranging from 0.7 to 0.5 while hypo-methylation ranging from 0.3 to 0.25.

### 3.5 Immune infiltration analysis data

Recently many studies have demonstrated that the tumor microenvironment (TME) is involved in the pathogensis and treatment of tumors [31,32]. As a basic component of TME, immune cells and cancer-associated fibroblast (CAF) are related to immune regulation and response in cancers. Hence, we applied different algorithms to investigate the association of CD244 expression with the level of infiltration of immune cell in various tumors, aimed at confiming the role of CD244 in TME. We discovered a strong positive correlation of CD244 expression with immune infiltrations of neutrophils, dendritic cells, monocytes, B cells, and CD8^+^ T cells across many tumor types (Fig. 4A). Meanwhile, data from the TISIDB database confirmed the positive association between the infiltration of CD8^+^ T cells and CD244 expression across multiple tumor types (Fig. 4B–H). In contrast, no negative correlation was discovered. It is worth noting that there was no correlation between CD244 expression and immune infiltrations of macrophages and NK cells across all TCGA tumor types (Supplementary Fig. 5).

**Fig. 4:**
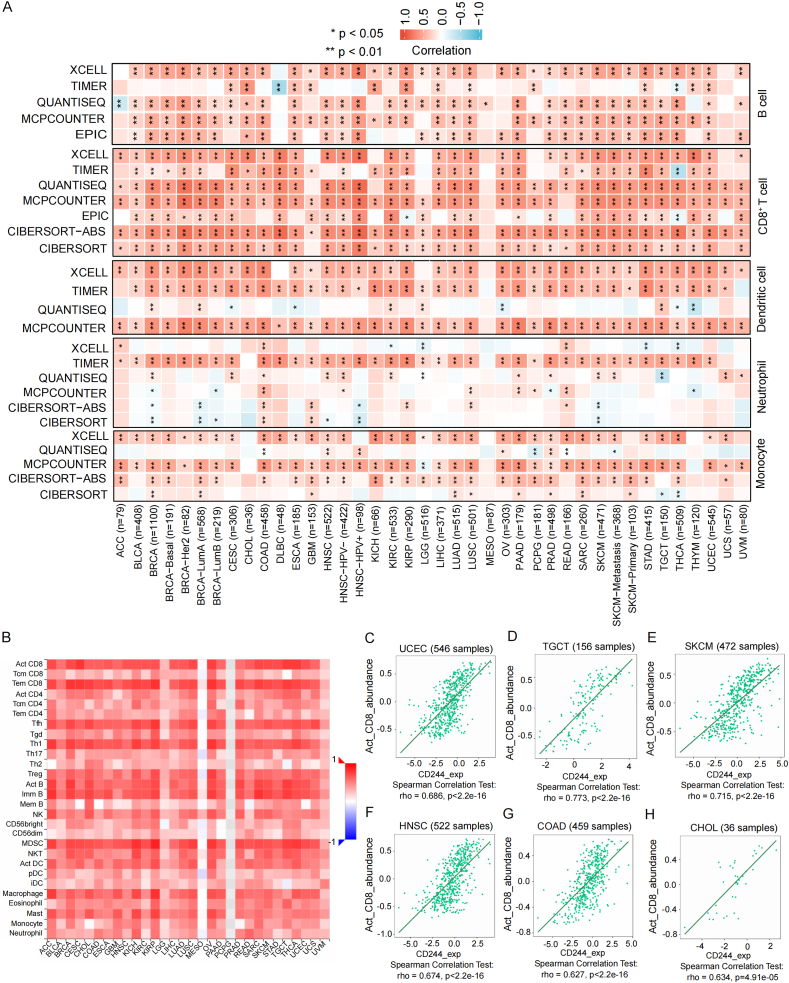
Relationships between CD244 expression and immune infiltration across various tumors. (A) The algorithms, including EPIC, MCPCOUNTER, QUANTISEQ, TIMER, XCELL, CIBERSORT, CIBERSORT-ABS, and TIDE, were utilized. The red color signals a positive correlation (0–1), and the blue color shows a negative correlation (−1–0). The correlation coefficient with a p < 0.05 is statistically significant, and statistically non-significant ones are marked with a cross. (B–H) The plot from the TISIDB database shows that CD244 is positively correlated with various kinds of immune cells across cancers.

To extend the knowledge of CD244 expression with immune infiltration, we further observed positive correlations between various lymphocytes infiltrating and the expression of CD244 in UCEC, especially the infiltrating of cytotoxic cells (Fig. 5A–E). We then investigated the relationship between CD244 expression and immunomodulatory molecules using TISIDB and GEPIA2 databases. A strong positive correlation was detected between CD244 expression and immunomodulators including CD96 (rho = 0.759), CTLA4 (rho = 0.636), HAVCR2 (rho = 0.706), TIGIT (rho = 0.688), CD247 (rho = 0.77), and PDCD1 (rho = 0.615) (Fig. 5F–J). Moreover, at single cell level, we confirmed the relationships between CD244 expression and the distribution of immune cells in UCEC, including CD8^+^ T cell (Fig. 5K-L). In addition, the expression level of CD244 in UCEC indicated by IHC was lower than that in normal tissues and positively associated with CD8^+^ T cell infiltration (Fig. 5M − O). These findings implied that CD244 is vital in regulating the immune cell infiltration.

**Fig. 5:**
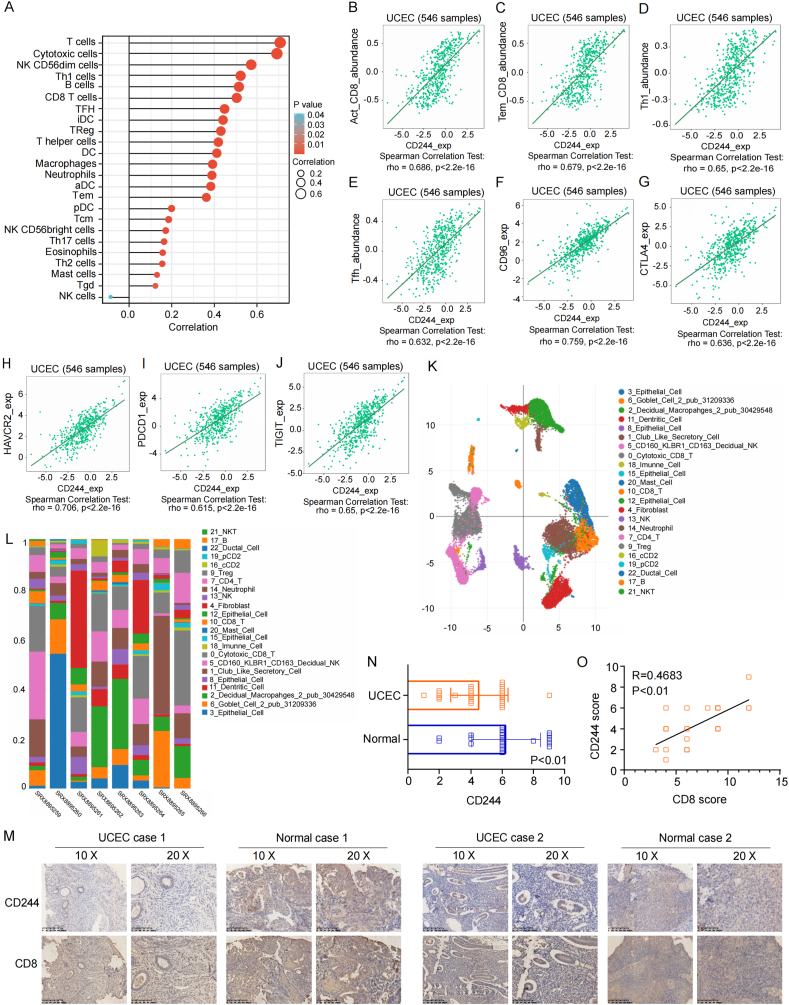
Correlations of CD244 expression with the immunocytes and immunomodulatory molecules in UCEC. (A) Associations between CD244 expression and various lymphocytes infiltrating in UCEC. (B–E) Associations between CD244 and various T cell subtypes. (F–J) Associations between CD244 expression and immunomodulators with a correlation coefficient of more than 0.6. (K) The SNE plot shows the distribution of various immune cells in UCEC at the single cell level. (L) The diagram represents the percentage of various immune cells in UCEC samples. (M) Immunohistochemical staining for CD244 and CD8 in normal and UCEC tissues. (N) CD244 expression levels in UCEC and controlled normal samples. (O) Correlation of CD244 with CD8^+^ T cell marker in expression.

Next, we used BEST database to explore the relationship between CD244 expression and immunotherapy response in cancer patients. As shown in Supplementary Fig. 6A–D, high expression levels of CD244 were positively associated with immunotherapy response in non-small cell lung cancer, melanoma and hepatocellular carcinoma. CD244 levels owned the diagnostic performance in discerning immunotherapy responders in melanoma (Supplementary Fig. 6E), non-small cell lung cancer (Supplementary Fig. 6F–G) and hepatocellular carcinoma (Supplementary Fig. 6H). In addition, after immunotherapy, the patients with high CD244 levels have a good progress-free survival (Supplementary Fig. 6I–J).

### 3.6 Single-cell sequencing analysis data

**Fig. 6:**
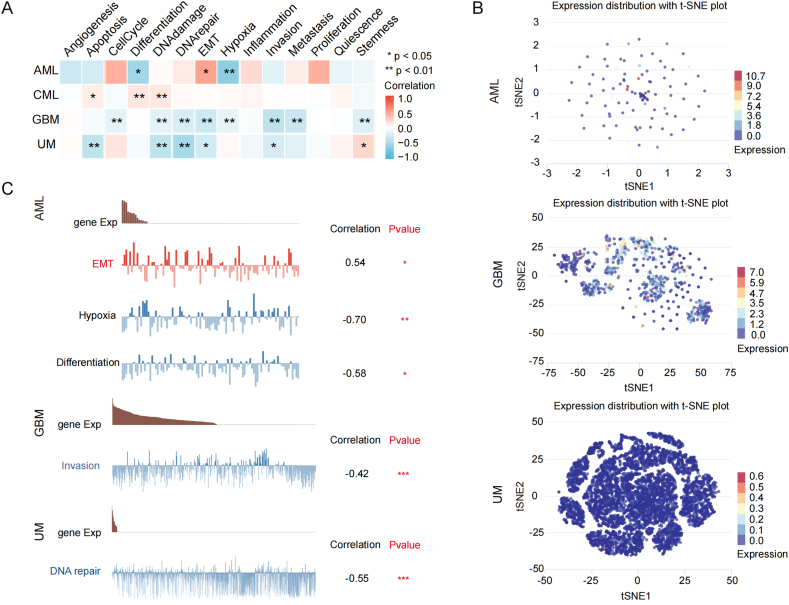
CD244 expression in single-cell level and association with functional status in different cancers. (A) The heat-map indicates the relations between the CD244 expression and functional states of tumors according to the CancerSEA database (**p < 0.01; *p < 0.05). (B) Functional states associated with CD244 gene expression are displayed (**p < 0.01; *p < 0.05). (C) T-SNE plot depicts the expression profile of CD244 in single cell of AML, GBM, and UM samples. The color of the dot represents its distinct expression levels.

Single-cell sequencing offers an opportunity to decode the cellular and molecular functional states of diverse cells. Its usage in cancer research promotes the interpretation of biological characteristics and dynamics of cancer cells [33,34]. The CancerSEA database is based on single-cell sequencing technology. We exploited it to certify the relevance of CD244 expression in 14 functional states in the single cell of distinct cancers. We discovered that CD244 expression in AML (acute myeloid leukemia) was not only significantly negatively associated with differentiation and hypoxia but was also positively associated with EMT (epithelial-mesenchymal transition). In addition, CD244 expression in GBM (glioblastoma) was significantly and negatively associated with invasion, and CD244 expression in UM (uveal melanoma) was significantly negatively associated with DNA repair (Fig. 6A). We also found the significant relevance between CD244 expression and EMT, hypoxia, and differentiation in AML; CD244 expression and invasion in GBM; and CD244 expression and DNA repair (Fig. 6B). Fig. 6C showed the distribution of CD244 expression in the single cell of AML, GBM, and UM. These findings imply that CD244 is essential in the pathogensis of tumors.

### 3.7 CD244 related gene functional enrichment results

**Fig. 7:**
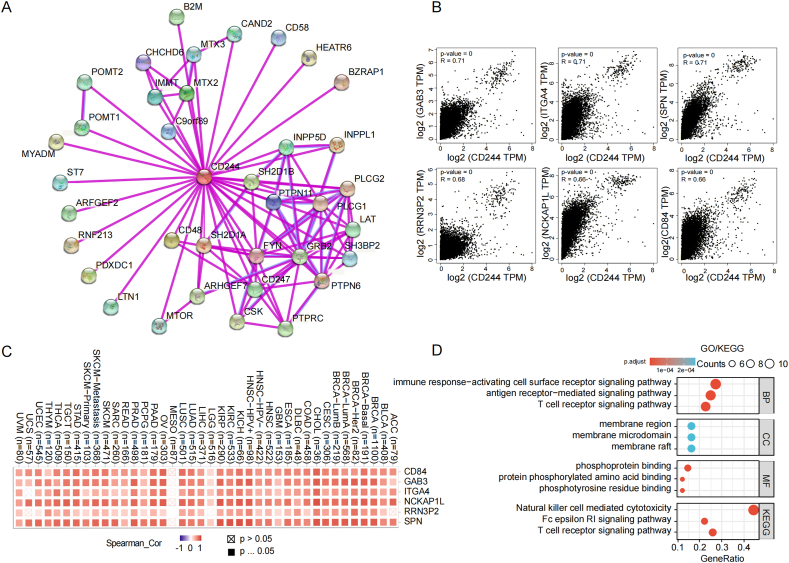
Gene enrichments and pathways analysis related to CD244. (A) The plot experimentally identified proteins for the top50 interaction, binding to CD244 or interacting with CD244. The edges show protein-protein interactions, and network nodes represent the individual proteins. (B) Relevance between CD244 and representative genes (GAB3, ITGA4, SPN, RRN3P2, NCKAP1L, and CD84) of the top CD244-associated genes from the TCGA project was assessed using GEPIA2. (C) The heat-map demonstrates the expression relevance between CD244 and GAB3, ITGA4, SPN, RRN3P2, NCKAP1L, and CD84 in TCGA cancers. (D) GO|KEGG analysis was based on the genes that bind to or interact with CD244.

In an attempt to explore the biological function of CD244 among various tumor tissues, we collected CD244-binding proteins and CD244 co-expression genes to analyze functional enrichments and signaling pathways. Depending on the STRING tool, we obtained the top 50 protein-protein interaction networks of CD244, including 35 kinds of proteins (Fig. 7A). Then, we acquired the top 100 CD244 expression-related genes using GEPIA2. The results revealed that the expression of CD244 had a strongly positive association with the expression of Grb3-associated-binder (GAB3) (R0.71), integrin alpha4 (ITGA4) (R0.71), sialophorin (SPN) (R0.71), RNA polymerase Ⅰ transcription factor pseudogene 2 (RRN3P2) (R0.68), NCK-associated protein 1 like (NCKAP1L) (R0.66), and leukocyte differentiation antigen 84 (CD84) (R0.66) (Fig. 7B). The heatmap data also displayed a positive association of CD244 with the aforementioned genes in most cancer types (Fig. 7C). Lastly, we conducted the GO and KEGG analysis on the previously collected CD244-interacting proteins and the CD244 expression related genes. The findings indicated that CD244 might exert vital functions in the biological processes of the natural killer cell mediated cytotoxicity, the immune response activating cell surface receptor signaling pathway, the antigen receptor mediated signaling pathway, and the T cell receptor signaling pathway (Fig. 7D).

## 4 Discussion

Identifying the role of cancer-associated genes is critically important to better understand oncogenic mechanisms, discover novel biomarkers, and develop efficient therapeutic agents. The pan-cancer analysis project creates an excellent opportunity to explore the molecular aberrations and their functions at the DNA, RNA, epigenetic, and protein levels across different tumor types [35]. This will guide us to uncover optimal biomarkers for the diagnosis, prognosis and effective treatments of a tumor type with similar genomic profiles.

Previous studies have demonstrated that CD244 acts as a cell surface receptor in immune response and regulation, and is involved in a series of biological processes in the pathogensis of cancer. CD244 is involved in immune tolerance and regulation of tumor cells via T cell inhibition [36]. Blocking CD244 on NK cells markedly attenuates the NK cell dysfunction induced by tumor-derived monocytes [37]. CD244 is increasingly expressed on exhausted CD8^+^ T cells with decreased production of IL-2 and IFN-γ in both mouse and human cancers [38]. Some T cell exhaustion markers like CD244 are potential prognostic biomarkers and therapeutic targets in colorectal cancer [39]. As a regulatory receptor expressed on many immune cells, CD244 can be considered as an immune checkpoint used for CAR-NK immunotherapy by constructing one chimeric antigen receptor (CAR) on NK cells [14]. Despite all the afore-mentioned advancements that have been made, a comprehensive assessment of the role of CD244 in development of cancer types remains unclear. Our study aimed at determining the effect of CD244 in the tumorigenesis and progression across various tumor types. Based on the datasets collected in the TCGA, GEO, and CPTAC databases, we systematically explored the CD244 gene expression, genetic alterations, promoter methylation, CD244-related prognosis and other molecular characterizations in 33 different tumor types.

We firstly compared the differences of CD244 expression in various tumor and normal tissues. Results showed that CD244 expression was lower in UCEC, COAD, LIHC, PRAD, LUAD, READ, and LUSC but was higher in KIRC and KIRP. We also investigated the promoter levels of CD244 across TCGA tumors, and found lower methylation levels of CD244 in UCEC tissues. In general, promoter hyper-methylation leads to the inhibition of gene expression [40]. However, emerging studies unexpectedly reported that promoter hypermethylation could also activate gene expression. For example, Wang et al. demonstrated both negative and positive correlations between gene expression and DNA methylation in rice genome [41]. Promoter methylated DNA can form anti-silencing complexes with some proteins that have specific domains binding to DNA; a possible mechanism by which promoter hyper-methylation contribute to enhanced gene expression. SUVH1 from *Arabidopsis thaliana*, a Su(var)3–9 homolog, could act as a transcriptional anti-silencing factor. Methylated DNA could be recognized by SUVH1, resulting in the improved gene expression [42,43]. Thus, these findings indicated complicated roles of CD244 methylation on its expression values.

The results of CD244 related survival analysis implied that high-expression levels of CD244 primarily served as a protective factor in most tumors. For example, an increased expression levels of CD244 indicated a better prognosis in ACC, SKCM, UCEC, CHOL, and HNSC. Strikingly, we noted a different survival outcome in HNSC compared with that from a previous study. CD244 was conducive to forming the immunosuppressive tumor microenvironment and the growth of HNSC in mice. By contrast, the intervention with monoclonal antibodies against CD244 caused remarkable growth -impaired of HNSC and increased the infiltration of CD8^+^ T cells [44]. The reason for this variation requires further investigation. Thus, CD244 is a potential biomarker for predicting the prognosis of many kinds of tumors.

The tumor cell's genome is full of abundant somatic mutations, most of which are driver mutations involved in tumor evolution and increase the risk of suffering from cancers [45]. Hence, we further explored the genetic alterations of CD244 to study its mutations and roles in cancers. We discovered that the highest alteration frequency of CD244 was above 15% in BLCA, and amplification and mutation are the primary types in all genetic alterations. Interestingly, our findings demonstrated a relationship between the CD244 genetic alterations and the survival rate of cancer patients with BRCA, COAD, and PCPG. Patients without CD244 genetic alterations have a better survival rate than their altered counterparts. This further illustrates that CD244 is a protective factor in oncogenesis and represents a potential biomarker for tumor prognosis.

Existing studies have reported the important roles of tumor microenvironment in the development and evolution of cancers [46]. CD244 represents a dual role in regulating immune responses through regulating the stimulatory or inhibitory signals [38,47]. CD244 expression on CD8^+^ T cells impaired the production of cytokines and increased apoptosis in mice with lung cancers [48]. Agresta et al. also reported increased expression of CD244 on several immune cells in a HNSC mouse model. CD244 activation significantly impaired the production of proinflammatory cytokines, subsequently inhibiting tumor growth [44]. Altvater et al. found that functioned as a costimulatory receptor, CD244 enhanced the tumor antigen-induced proliferation and activation of anti-tumor T cells [49]. Here, our results revealed a positive correlation between the expression of CD244 and the immune infiltrations of CD8^+^ T cells, B cells, dendritic cells, and monocytes in many cancer types, especially like UCEC.

The single-cell sequencing analysis suggested that CD244 expression was significantly and negatively associated with differentiation and hypoxia and positively associated with EMT (epithelial-mesenchymal transition) in AML. Moreover, CD244 expression was significantly and negatively associated with invasion in GBM, and was significantly and negatively associated with DNA repair in UM. Further research and validation are needed in each tumor to study the relationship between CD244 and the biological processes of EMT, differentiation, invasion, hypoxia, and DNA repair. The enrichment analysis of GO and KEGG pathways has been proved to predict and analyze the roles of essential genes in an organism's survival [50]. Our results revealed that CD244 is involved in the biological processes of the natural killer cell mediated cytotoxicity, the immune response activating cell surface receptor signaling pathway, the antigen receptor mediated signaling pathway, and the T cell receptor signaling pathway. Altogether, CD244 plays an inhibitory (or protective) role in the inition and progression of cancer.

## 5 Conclusion

In summary, our current study presents a complete overview of the molecular characteristics of CD244 and its biological function in the initiation and evolution of tumors. We found that CD244 expression was down-regulated in many cancers as well as significant associations between CD244 expression and clinical pathological stages, tumor prognosis, and tumor functional states in various human tumors. We also discovered a correlation between genetic alterations of CD244 and clinical prognosis in several cancers. More significantly, IHC confirmed that the CD244 expression level in normal tissues was higher than that in UCEC tissues and detected a positive correlation of CD244 expression with CD8^+^ T cells infiltrating in UCEC. Our findings indicate that CD244 plays a protective role in tumorigenesis, as well as prognostic and therapeutic roles in human tumors.

## Funding statement

No funding was supported for this research work.

## Data availability statement

All raw data can be access from the corresponding author on reasonable request.

## CRediT authorship contribution statement

**Zhenzhen Deng:** Methodology, Investigation, Data curation. **Yuanhong Liu:** Methodology, Investigation, Conceptualization. **Haiyan Zhou:** Writing – review & editing, Writing – original draft, Visualization, Validation.

## Declaration of competing interest

The authors declare that they have no known competing financial interests or personal relationships that could have appeared to influence the work reported in this paper.

## Figures and Tables

**figs1:**
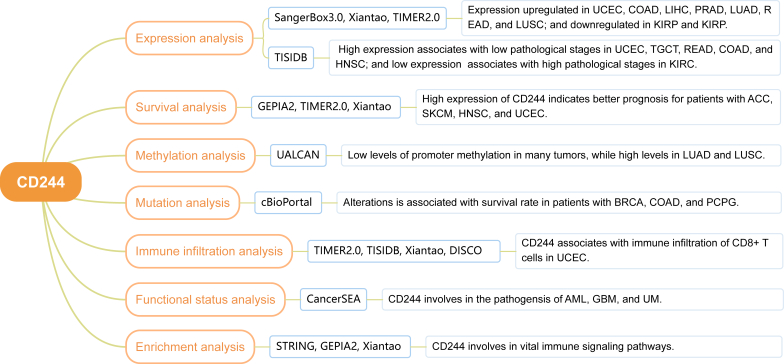


**figs2:**
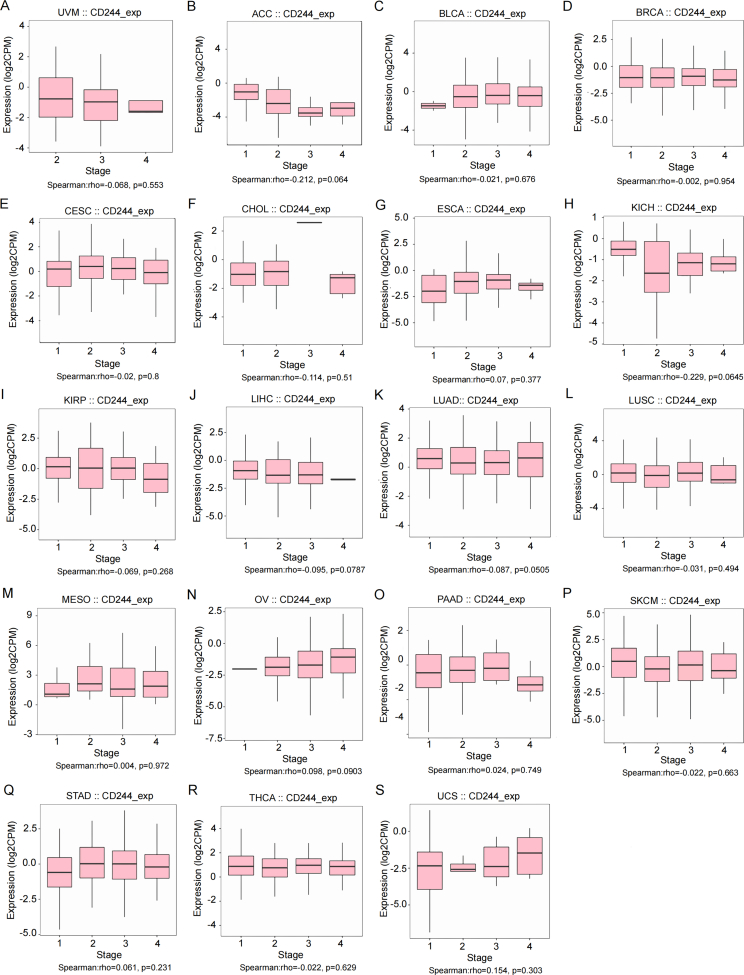


**figs3:**
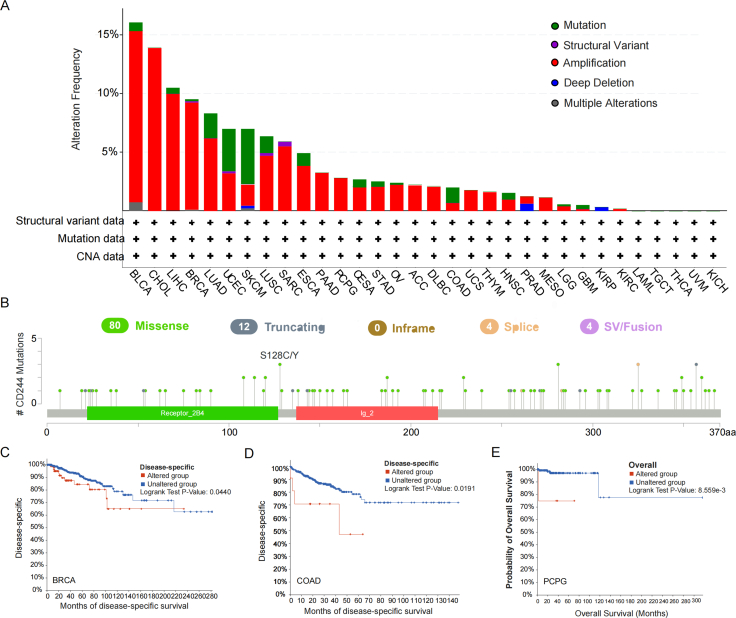


**figs4:**
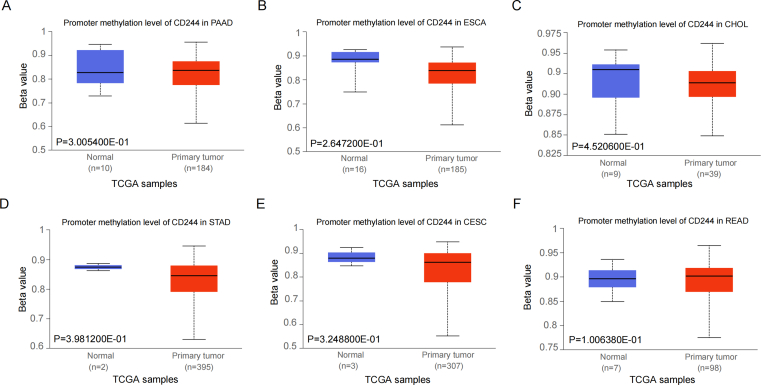


**figs5:**
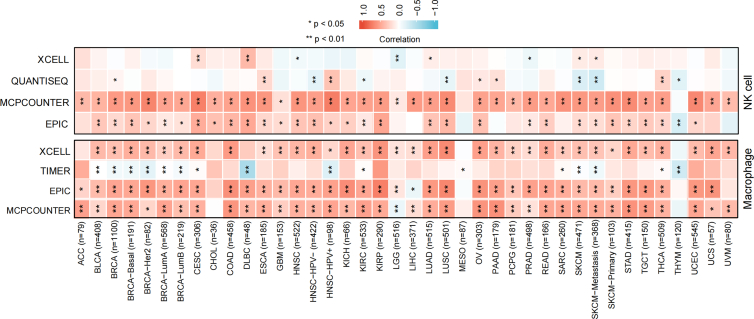


**figs6:**